# Impact of Body Composition on Progression-Free Survival in Patients with Metastatic Breast Cancer Treated with Ribociclib

**DOI:** 10.3390/curroncol32090510

**Published:** 2025-09-13

**Authors:** Ahmet Oruç, Mustafa Erol, Özlem Şahin, Melek Karakurt Eryılmaz, Murat Araz, Mehmet Artaç

**Affiliations:** 1 Clinic of Medical Oncology, Faculty of Medicine, Necmettin Erbakan University, Konya 42090, Turkey; mkeryilmaz@erbakan.edu.tr (M.K.E.); maraz@erbakan.edu.tr (M.A.); martac@erbakan.edu.tr (M.A.); 2 Department of Nuclear Medicine, Faculty of Medicine, Necmettin Erbakan University, Konya 42090, Turkey; merol@erbakan.edu.tr (M.E.); ozlemsahin@erbakan.edu.tr (Ö.Ş.)

**Keywords:** ribociclib, SAT, VAT, AMG, SMI

## Abstract

Purpose: This study aims to determine whether body composition parameters affect progression-free survival (PFS) in patients with hormone receptor positive and HER-2 negative metastatic breast cancer treated with ribociclib as first-line therapy. Materials and methods: It was designed as a single-center, retrospective study; therefore, its generalizability is limited. At the start of treatment, ^18^F-FDG PET/CT scans were performed on patients, and subcutaneous adipose tissue (SAT) and visceral adipose tissue (VAT) volume, SAT and VAT SUV (standardized uptake value) mean, SAT and VAT index, skeletal muscle index (SMI), and skeletal muscle radiodensity (SMD) were calculated at the L3 vertebra level. The albumin-myosteatosis gauge (AMG) was defined as SMD × albumin. Results: The study included 73 participants. Increased SAT and VAT volumes were associated with worse PFS (23.4 vs. 35.5 months, *p*: 0.015; 25.4 vs. 33.3 months, *p*: 0.114). However, in the multivariable cox regression analysis for progression free survival (PFS), an increase in SAT volume [HR 4.96; *p*: 0.038)] and SAT SUV mean [HR 2.99; *p*: 0.016)] were identified as independent risk factors. Conclusions: It should be noted that in patients treated with ribociclib, increases in SAT volume and SAT SUV mean are independent risk factors for PFS.

## Simple Summary

Ribociclib is an oral agent used as first-line treatment in hormone receptor-positive, HER-2-negative metastatic breast cancer. Previous studies have shown that body mass index does not affect treatment efficacy. In this study, we investigated whether waist circumference, body fat measurements, and muscle mass differences affect treatment benefit. The results showed that patients with the same weight but higher subcutaneous fat tissue volume were unable to continue ribociclib treatment due to early disease progression or death compared to the group with lower volume.

## 1. Introduction

Breast cancer is the most common type of cancer in women (23.8%) and 2,296,840 new cases were diagnosed worldwide in 2022. However, when both sexes are considered, it is the third leading cause of death among all cancers [1]. The most common subtype of breast cancer (70%) is hormone receptor-positive (HR) and HER-2 (human epidermal growth factor receptor 2) negative [2]. This subtype is important because, despite being less aggressive and growing more slowly than triple-negative and HER-2 positive subtypes, it is more common. First-line treatment for this subgroup consists of CDK4/6 (cyclin-dependent kinase) inhibitors combined with aromatase inhibitors or fulvestrant [3]. Ribociclib is the only drug in this class that has demonstrated benefits in both progression-free survival (PFS) and overall survival (OS) [4].

Studies investigating the effect of obesity and excess weight on breast cancer survival have yielded conflicting results. Although it has been shown to negatively affect survival in early-stage breast cancer, studies have found that being overweight and obese are not associated with poorer outcomes in women with metastatic disease [5,6,7]. On the other hand, there are very few studies investigating the relationship between body mass index (BMI) and CDK4/6 inhibitors used in first-line treatment in this patient group. A recent study demonstrated that the addition of abemaciclib to hormone therapy in metastatic HR-positive HER-2-negative breast cancer extended progression-free survival (PFS) independently of BMI and provided benefits in overweight or obese patients as well [8].

BMI and weight are not adequate indicators of fatness. This is because they do not distinguish between muscle and fat tissue, nor do they distinguish between specific parts of fat tissue that have different physiological effects, such as visceral and subcutaneous fat [9]. Since BMI is not considered a prognostic factor for survival in breast cancer, subcutaneous adipose tissue (SAT) and visceral adipose tissue (VAT) have been evaluated as prognostic factors in studies. As a result, SAT volume and VAT volume were examined in non-metastatic breast cancer, and it was found that an increase in SAT volume increases the risk of death [10]. Furthermore, an increase in the mean VAT SUV (standardized uptake value) measured by ^18^F-fluorodeoxyglucose positron emission tomography/computed tomography (^18^F-FDG PET/CT) in early-stage breast cancer is associated with poorer recurrence-free survival (RFS) and distant metastasis-free survival (DMFS) [11]. High density detected in SAT and VAT has been found to increase the risk of overall mortality [12].

Several studies have indicated that sarcopenia is associated with poor survival in metastatic breast cancer and that skeletal muscle index (SMI) may be an indicator of this [13]. On the other hand, given the complex interaction between systemic inflammation, poor nutritional status, and muscle loss in cancer progression, it is important to integrate albumin and myosteatosis into a single prognostic indicator. The Albumin-Myosteatosis Gauge (AMG) proposed for this purpose has previously been studied for its ability to determine prognosis in cancer patients, even in non-breast cancers. The objective of these studies was to investigate the relationship between albumin, which serves as both a negative acute phase reactant and an indicator of nutritional status, and abdominal circumference muscle tissue density [14,15]. In these studies, patients with low AMG levels were found to have lower survival rates and poorer treatment responses.

This study was designed to evaluate the relationship between BMI, adipose tissue parameters, and SMI, SMD, and AMG with PFS in patients with metastatic HR positive HER-2 negative breast cancer treated with ribociclib in the first line.

## 2. Material and Methods

### 2.1. Patients and Survival

The clinical and pathological characteristics of the patients were obtained from the hospital database system. The study included patients who started ribociclib treatment between September 2020 and January 2025, had PET/CT scans at baseline, had no metastases at L3 level, had no second primary cancer, had not received chemotherapy prior to ribociclib treatment, and were over 18 years of age.

Progression-free survival (PFS) was defined as the time to progression under ribociclib or death if death occurred under treatment. In patients without progression, PFS was calculated as the time until data entry.

### 2.2. Body Composition Analysis

**Figure 1:**
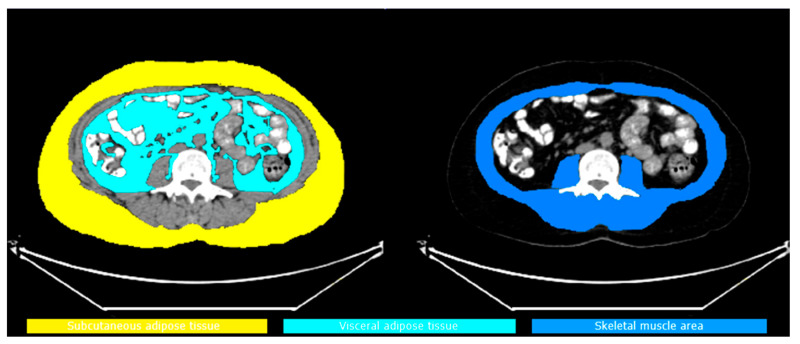
SAT, VAT and SMI areas.

Imaging was performed using the Siemens Biograph 6 TruePoint ^18^F-FDG PET/CT (Siemens, Germany) device, with a whole-body scan from the vertex to the mid-femur level. Low-dose CT (120 kVp, 50 mAs) was used for attenuation correction and anatomical localization. Volume measurements and SUV mean values were calculated using regions of interest (ROIs) drawn on CT slices in the axial plane, which were automatically integrated into the ^18^F-FDG PET/CT fusion images. Subcutaneous fat tissue (SAT) and visceral fat tissue (VAT) volumes were calculated from five consecutive axial slices at the L3 vertebra (L3) level. SAT is defined as the fat tissue from the outer border of the abdominal wall to the muscle layer; VAT is defined as the fat tissue from the inner border of the muscle layer to the peritoneal cavity (as shown Figure 1). SAT SUVmean and VAT SUVmean were calculated based on the average SUV values in 5 sections. The body composition parameters were calculated by squaring the height in meters and were defined as indexes encompassing the skeletal muscle index (SMI), SAT index and VAT index. The SMD was computed as the mean Hounsfield unit (HU) value of the SMA. The cut-off value of 41 used in the study by Martin et al. was adopted for SMI [16]. We computed the AMG according to the following formula: AMG = SMD (HU) × serum albumin (g/dL), as suggested by Kim et al. [14]. To keep things simple, an arbitrary unit was used instead of HU × g/dL as the AMG unit.

For accurate metabolic activity measurement, ROIs were carefully evaluated and manually excluded to prevent high ^18^F-FDG FDG uptake from adjacent tissues (e.g., liver, intestines, vascular structures, kidneys, ureters, and muscles). This process was jointly performed by two nuclear medicine specialists, and average values were used to minimize inter-observer variation in measurements (kappa coefficient: 0.89, *p* < 0.001).

### 2.3. Statistical Analysis

The Kolmogorov–Smirnov test was used to evaluate the normality assumption for continuous variables. For data that did not demonstrate a normal distribution, the Mann–Whitney U test was used to compare groups, while the chi-squared test was used for categorical variables. We presented categorical variables as frequency (*n*) and percentage (%). Kaplan–Meier survival curve was used to calculate survival, and the log-rank test was used to compare differences in survival time. All variables affecting prognosis and PFS were evaluated first by univariate and then by multivariate cox regression analysis. Multivariate Cox regression analysis was performed using the backward LR method, with *p*-values < 0.25 were included. The effects of the variables included in the regression model were expressed as hazard ratios (HRs) with 95% confidence intervals (CIs). All statistical analyses were performed using IBM SPSS Statistics 25.0 (Armonk, NY, USA). Two-sided *p*-values < 0.05 were considered statistically significant.

### 2.4. Ethics Approval

This study was conducted in accordance with the principles of the Helsinki Declaration. The Ethics Committee of Necmettin Erbakan University approved the study (Date: 9 May 2025/No: 2025/5766). The informed consent was waived due to the retrospective nature of the study.

## 3. Results

### 3.1. Study Population and Characteristics

**Figure 2:**
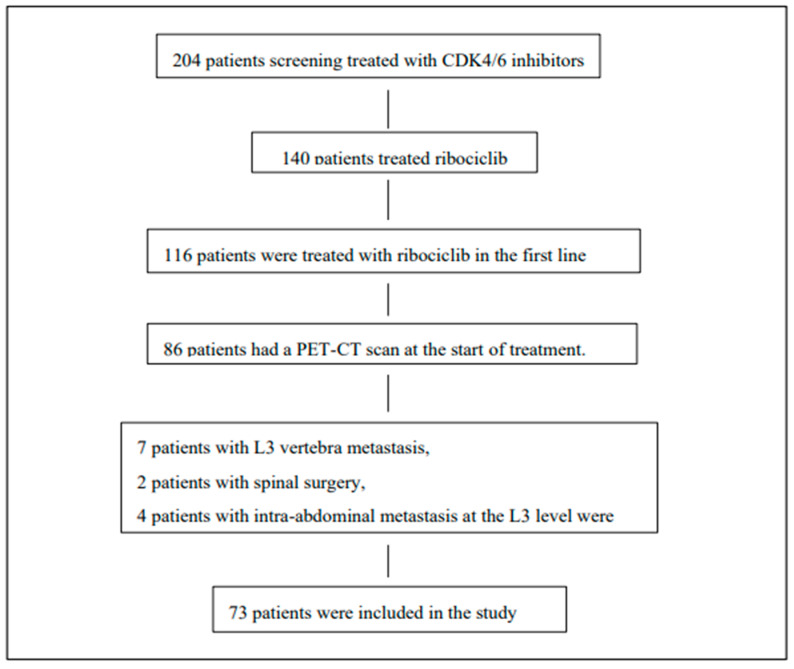
Study population: eligibility criteria and reasons for exclusion of patients from the study.

The study included 73 patients with HR positive, HER-2 negative metastatic breast cancer who received first-line ribociclib therapy. The inclusion and exclusion criteria for the screened patients are shown in Figure 2.

As shown in Table 1, the median age (interquartile range) was 56 (44–68.5), and 34 (46.5%) patients had de novo metastatic disease. The median BMI (IQR) was 28.05 (25.3–31.2), the most common pathological subtype was invasive ductal carcinoma (61, 83.6%), and the most common site of metastasis was bone metastasis, detected in 57 (78%) patients.

### 3.2. Body Composition Parameters and Survival

**Figure 3:**
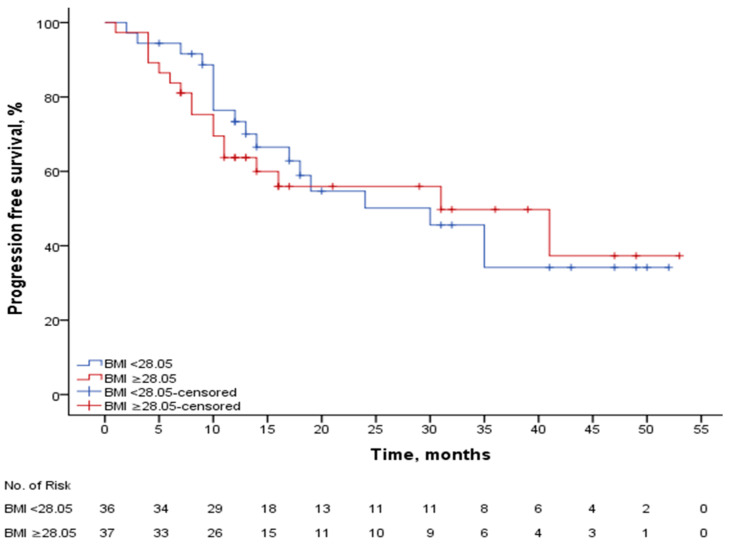
PFS according to median BMI.

The median follow-up period was 22 months, and overall survival maturity has not been established. As shown in Figure 3, the median(m) PFS was 30.4 (22.9–37.9) months in patients with a BMI of 28.05 and above (n: 37) and 29.4 (22.6–36.2) months in patients with a BMI below 28.05 (n: 36), *p*: 0.834. When BMI was set at 25, mPFS was 31 (24.9–37.0) months in patients with a BMI ≥ 25 (n: 55), while mPFS was 26.5 (18.6–34.4) months in patients with a BMI < 25 (n: 18), *p*: 0.869.

**Figure 4:**
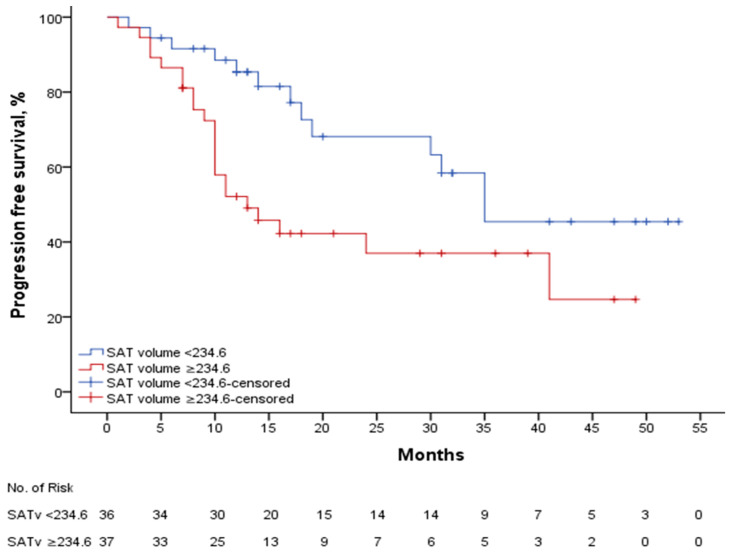
PFS according to SAT volume.

The median SAT SUV mean was 0.27, with a mPFS of 28.1 (21.4–34.8) months in patients with a SAT SUV mean ≥ 0.27 (n: 39), while the mPFS was 32.4 (24.6–40.2) months in patients with a SAT SUV mean < 0.27 (n: 34), *p*: 0.454. In patients with a SAT volume of ≥234.6 (n: 37), the median mPFS was 23.4 (17–29.8) months, while in patients with a SAT volume < 234.6 (n: 36), the median PFS was 35.5 (28.5–42.5) months, *p*: 0.015. Figure 4. shows the PFS curve according to SAT volume. In patients with SAT volume ≥ 234.6, the median mPFS was 21.1 (14.9–27.4) months in those treated with ribociclib plus letrozole (n: 20), compared to 20.5 (11.6–29.3) months in those treated with fulvestrant (n: 17), *p*: 0.335. In patients with SAT volume < 234.6, the median progression-free survival (mPFS) was 32.9 (24–41.8) months in those treated with letrozole in combination with ribociclib (n: 25) and 38.2 (27–49.4) months in those treated with fulvestrant in combination (n: 11), *p*: 0.594.

The median SAT index was 90.19, with mPFS of 25.1 (18.4–31.8) months in patients with a SAT index of ≥ 90.19 (n: 37), while mPFS was 33.9 (26.8–41) months in patients with a SAT index < 90.19 (n: 36), *p*: 0.066.

The median VAT SUV mean was 0.72. In patients with a VAT SUV mean of ≥ 0.72 (n: 39), the mPFS was 32.1 (25.5–38.7) months, while in patients with a VAT SUV < 0.72 (n: 34), the mPFS was 26.1 (18.3–33.9) months, *p*: 0.332. The median VAT volume was 132.3, and in patients with a VAT volume of ≥ 132.3 (n: 37), the median mPFS was 25.4 (18.5–32.3) months, while in patients with a VAT volume < 132.3 (n: 36), the median PFS was 33.3 (26.5–40.1) months, *p*: 0.114. The median VAT index was determined to be 52.8. In patients with a VAT index of ≥52.8 (n: 37), mPFS was 25.6 (18.9–32.3) months, while in patients with a VAT index < 52.8 (n: 36), the median PFS was 32.8 (25.8–39.8) months, *p*: 0.171.

In patients with ≥SMI 41 (n: 60), the median PFS was 30.2 months (24.5–35.9), while in patients with SMI < 41 (n: 13), the median PFS was 26.4 months (15.8–37), *p*: 0.832. The median SMD was 31. In patients with SMD ≥ 31 (n: 39), the median PFS was 31.2 (24–38.4) months, and in patients with SMD < 31 (n: 34), the median PFS was 27.5 (20.7–34.3) months, *p* = 0.546. The median albumin myosteatosis gauge was 135.5. Patients with AMG ≥ 135.5 (n: 35) had a median PFS of 30.2 (22.8–37.7) months, while patients with AMG < 135.5 (n: 33) had a median PFS of 27.8 (20.7–35) months, *p*: 0.720.

BMI, SAT suv mean, SAT volume, SAT index and VAT suvmean, VAT volume, VAT index and SMI, SMD and, were first evaluated using univariate cox regression analysis for progression. Multivariate Cox regression analysis was performed using SAT volume, SAT SUV mean, and SAT index. The results of the cox regression analysis are presented in Table 2. As a result, the HR (hazard ratio) for SAT suv mean was found to be 3.15 (1.06–9.32: 0.038) and the HR for SAT volume was found to be 4.96 (1.34–18.2: 0.016). Sensitivity analysis was performed with BMI and age. In the analysis with BMI, the HR for SAT volume was 6.2 (CI: 1.7–22.9, *p*: 0.05) and for SAT SUV mean was 3.1 (1.08–8.9, *p*: 0.035). In the analysis by age, HR was 3.1 (1.1–8.7, *p*: 0.024) for SAT volume and HR was 2.6 (0.9–7.3, *p*: 0.057) for SAT SUV mean.

### 3.3. Body Composition Parameters and Treatment-Related Toxicity

At least one adverse effect of any grade developed during treatment in 27 patients. A permanent dose reduction was also performed in 21 patients. As shown Table 3, there is a relationship between body parameters and adverse effects and dose reduction.

The incidence of treatment-related adverse effects is higher in patients with a SAT SUV mean <0.27 and a VAT SUV mean <0.72. Permanent dose reduction is not affected by body composition parameters and is only higher in patients aged 65 years and older. Detailed data on adverse effects associated with SAT suv mean and VAT suv mean are shown in Tables S1 and S2.

## 4. Discussion

In this cohort of 73 patients with HR positive and HER-2 negative breast cancer treated with ribociclib, an increase in SAT volume and an increase in SAT SUV mean were found to be independent risk factors for progression in patients with metastatic breast cancer. The absence of differences between groups in terms of BMI, but changes in SAT and VAT measurements, demonstrated that these two parameters are more effective than BMI. Although previous studies have examined volumetric changes in fat distribution and SMI with CDK4/6 inhibitors in a limited number of patients, our study is important to comprehensively investigate AMG and SUV mean values in this context.

Obesity is associated with an increased incidence and progression of many types of cancer and is estimated to contribute to up to 20% of cancer-related deaths [17]. Metabolic and inflammatory changes in adipose tissue, which disrupt physiological homeostasis both locally and systemically, are responsible for this. Cancer-associated adipocytes (CAAs), inflammation in adipose tissue, increased IL-6 (interleukin-6) and TNF (tumor necrosis factor) levels, and changes in adiponectin levels are known mechanisms [18]. White adipose tissue has an increase in the number of mast cells [19] and macrophages [20] that secrete proinflammatory cytokines. The systemic release of proinflammatory cytokines triggers chronic low-grade inflammation, which leads to the production of reactive oxygen species and tumor progression. Therefore, SUV mean measured in adipose tissue may serve as a non-invasive marker of systemic inflammation and a prognostic indicator in breast cancer patients [11]. In recent years, it has been demonstrated that changes in metabolic and inflammatory pathways associated with adipose tissue microenvironment (ATME) also contribute to the development and progression of cancer [21].

Until now, studies examining the effect of obesity on breast cancer risk or cancer-related outcomes have used BMI as an indicator of obesity. While BMI is a simple and inexpensive indicator of obesity, it does not provide information about body fat distribution or muscle mass. As BMI is independent of age, it is unable to predict the impact of menopausal status on breast cancer [22]. The absence of a difference in PFS between BMI groups in our study is consistent with previous studies and is important in that it demonstrates that BMI is not a prognostic factor. Therefore, although not all of these factors were evaluated together in studies investigating body fat distribution and its effect on prognosis in breast cancer, SAT and VAT were calculated in terms of volume, density, SAT/VAT ratio and SUV value [23,24,25]. One of these studies provided strong evidence that VAT/SAT ratio and VAT metabolic activity assessed by preoperative ^18^F-FDG PET/CT may predict axillary lymph node (ALN) metastasis in postmenopausal patients with luminal breast cancer [25]. Another study also indicated that the SAT index may predict bone metastasis in metastatic breast cancer [26].

Ribociclib undergoes extensive hepatic metabolism in humans, primarily via CYP3A4. It is administered at a dose of 600 mg per day and is independent of weight or body surface area. Although the majority is eliminated via the liver and subsequently via feces and urine, its relationship with adipose tissue is not fully understood [27]. In a study examining trastuzumab deruxtecan and body fat distribution in metastatic breast cancer, a relationship was found between dose reduction and high SAT and VAT, and a lower objective response rate was obtained in patients with high SAT [24]. Similarly to this study, our study also demonstrated that an increase in SAT volume is associated with PFS. However, when examining the side effects associated with ribociclib, we believe that the decrease in both SAT suv mean and VAT suv mean is significant in relation to the increased incidence of ribociclib-related side effects. It has been suggested that some Cytochromes P450 enzymes previously found in adipose tissue are active in adipose tissue rather than in the liver, and that this may result in a relationship between drug efficacy and elimination and adipose tissue levels [28]. Considering that the increase in SAT SUV mean poor PFS and that there are fewer side effects, it is necessary to investigate the distribution of ribociclib in adipose tissue. We believe that the probable cause is increased inflammation in adipose tissue and a consequent increase in the SUV mean. This increases drug metabolism, reducing the drug’s effect and consequently leading to a reduction in side effects. Although VAT SUV mean showed numerically better PFS in our study, the difference was not statistically significant. An increase in SAT SUV mean was found to negatively affect PFS, supporting a previous study showing that preoperative VAT SUV mean in early-stage breast cancer is associated with poor RFS and DMSF [11]. Further, more comprehensive studies are needed in this area.

Both the association between SAT volume increase and poor PFS and the association between increasing SAT suv mean and side effects may guide clinicians in their decision-making process at the start of treatment. At the very least, patients should be advised to adopt a diet and lifestyle changes that reduce SAT volume, or, if this is not possible, professional support should be offered.

There are a limited number of studies investigating the survival of CDK4/6 inhibitors about BMI. No difference in survival based on BMI was found in patients receiving abemaciclib plus endocrine therapy [8]. In another study, although the median follow-up duration and number of patients were limited, and fewer patients in the subgroup used ribociclib compared to our study, PFS did not differ based on BMI. This supports the consistency of our findings [29]. This supports the consistency of our results.

There are very few studies in the literature evaluating body composition parameters and survival or response with CDK4/6 inhibitors. In these studies, a decrease in SMI reduced survival, which is similar to our study. However, it should be noted that the lack of effect of SAT and VAT increases on survival in both studies may be due to the very limited number of patients receiving ribociclib and the need to evaluate the study power accordingly [30,31]. Additionally, the possibility of differences between ribociclib and other CDK4/6 inhibitors should not be overlooked. Recently, 23 retrospective studies on SAT and VAT in breast cancer (involving 12,462 patients) have shown an association between increased SAT and VAT values and poor survival, consistent with our study [32]. Since VAT and SAT values are related to volume, but we also wanted to see their relationship with patients’ height, both SAT index and VAT index were calculated. An increase in both indices was found to be associated with poor PFS, which was similar to the largest study conducted on this subject [32].

Inflammation caused by the tumor and cancer cachexia is associated with myosteatosis and serum albumin levels. Studies have suggested that the inhibition of muscle proteolysis and hepatic albumin production is caused by increased production of proinflammatory mediators, such as IL-6. The tumor microenvironment releases proinflammatory cytokines, including TNF-α and IL-6 [33]. These cytokines may mediate the redistribution of adipose tissue and the infiltration of intramuscular fat by inducing muscle progenitor cells to differentiate into an adipocyte-like phenotype. Based on these findings, it can be concluded that myosteatosis and serum albumin levels reflect cachexia in different ways. Therefore, integrating these two factors may have a synergistic effect on prognosis stratification. To the best of our knowledge, this is the first study evaluating AMG and ribociclib in the literature. Similarly to previous studies, although increased AMG was associated with better PFS, the difference was not statistically significant. Nevertheless, cachexia and myosteatosis are important factors affecting survival in this patient group.

This study had several limitations. First, the small number of patients (n: 73) and the retrospective and observational nature of the study may have led to data loss and bias. Therefore, there is a possibility of type 1 error. Second, the relationship between adipose tissue and tumor biology in each patient may have affected the results. The possibility of collinearity between SAT volume, SAT index, and VAT measurements should be considered. Another limitation is that only patients who underwent PET/CT scanning at baseline were included in the study; the results of patients whose staging was performed using only computed tomography (CT) and bone scintigraphy should also have been evaluated. Validation in an independent cohort is required. However, to minimize bias, all eligible consecutive patients were included in the study. Prospective studies are needed to confirm the predictive role of SAT volume/SUV mean. Despite these limitations, this study is the first to include such a large group of patients with metastatic breast cancer receiving ribociclib.

## 5. Conclusions

In conclusion, it was determined that PFS was poor with increases in SAT and VAT volume, and SAT volume and SUV mean may serve as potential independent prognostic indicators.

## Figures and Tables

**Table 1:** Clinical and pathological characteristics.

	*n*: 73
Age, median (IQR)	56 (44–68.5)
De novo metastasis, *n* (%)	34 (46.5%)
Number of postmenopausal patients *n* (%)	35 (47.9%)
Body mass index, median (IQR)	28.05 (25.3–31.2%)
İnvasive ductal carcinoma, *n* (%)	61(83.6%)
İnvasive lobular carcinoma, *n* (%)	8 (10.9%)
İnvasive mixed carcinoma, *n* (%)	4 (5.5%)
Bone metastasis, *n* (%)	57 (78%)
Liver metastasis, *n* (%)	10 (13.6%)
Lung metastasis, *n* (%)	24 (32.8%)
Lymph node metastasis, *n* (%)	43 (58.9%)
Ribociclib with letrozole, *n* (%)	45 (61.6%)
Ribociclib with fulvestrant, *n* (%)	28 (38.4%)

**Table 2:** Cox regression analysis for PFS.

	Univariable Analysis HR (95% CI)	*p*	Multivariable Analysis HR (%95 CI)	*p*
Age (≥65 vs. <65)	1.28 (0.45–3.67)	0.635		
BMI (≥25 vs. <25 kg/m^2^)	1.35 (0.45–4.01)	0.590		
Menopausal status (+ vs. −)	0.72 (0.26–1.99)	0.528		
De- novo metastasis (+ vs. −)	1.33 (0.53–3.35)	0.541		
Endocrine partner drug	0.94 (0.34–2.60)	0.910		
SAT suvmean (≥0.27 vs. <0.27)	2.09 (0.81–5.33)	0.123	3.15 (1.06–9.32)	0.038
SAT volume (≥234.6 vs. <234.6	2.59 (1.008–6.67)	0.048	4.96 (1.34–18.2)	0.016
SAT index (≥90.1 vs. <90.1)	2.06 (0.81–5.24)	0.129	2.42 (0.22–26.0)	0.465
VAT suvmean (≥0.72 vs. <0.72)	0.93 (0.37–2.35)	0.887		
VAT volume (≥132 vs. <132)	1.32 (0.52–3.31)	0.555		
VAT index (≥52.8 vs. <52.8)	1.31 (0.52–3.31)	0.555		
SMI (≥41 vs. <41)	0.75 (0.22–2.49)	0.639		
SMD (≥31 vs. <31)	0.68 (0.27–1.73)	0.426		
AMG (≥135.5 vs. <135.5)	0.79 (0.30–2.05)	0.633		

**Table 3:** Toxicity and dose reduction.

*n*: 73	Adverse Events	*p*	Dose Reduction	*p*
Age (≥65 vs. <65)	9 (47.4%) vs. 18 (33.3%)	0.276	10 (52.6%) vs. 11 (20.4%)	0.008
BMI (≥25 vs. <25 kg/m^2^)	19 (34.5%) vs. 8 (47.1%)	0.352	15 (27.3%) vs. 6 (35.3%)	0.525
SAT suvmean (≥0.27 vs. <0.27)	9 (23.1%) vs. 18 (52.9%)	0.008	12 (30.8%) vs. 9 (26.5%)	0.686
SAT volume (≥234.6 vs. <234.6)	12 (32.4%) vs. 15 (41.7%)	0.414	11 (29.7%) vs. 10 (27.8%)	0.854
SAT index (≥90.1 vs. <90.1)	12 (32.4%) vs. 15 (41.7%)	0.414	10 (27%) vs. 11 (30.6%)	0.739
VAT suvmean (≥0.72 vs. <0.72)	10 (25.6%) vs. 17 (50%)	0.032	13 (33.3%) vs. 8 (23.5%)	0.356
VAT volume (≥132 vs. <132)	11 (29.7%) vs. 16 (44.4%)	0.193	9 (24.3%) vs. 12 (33.3%)	0.395
VAT index (≥52.8 vs. <52.8)	11 (29.7%) vs. 16 (44.4%)	0.193	9 (24.3%) vs. 12 (33.3%)	0.395
SMI (≥41 vs. <41)	21 (35.0%) vs. 6 (46.2%)	0.450	18 (30%) vs. 3 (23.1%)	0.617
SMD (≥31 vs. <31)	14 (35.9%) vs. 13 (38.2%)	0.836	8 (20.5%) vs. 13 (38.2%)	0.095
AMG (≥135.5 vs. <135.5)	13 (34.1%) vs. 12 (32.4%)	0.811	8 (21.6%) vs. 12 (32.4%)	0.222

## Data Availability

The data that support the findings of this study are available from the corresponding author upon reasonable request.

## Supplementary Materials

The following supporting information can be downloaded at: https://www.mdpi.com/article/10.3390/curroncol32090510/s1, Table S1: Adverse effects according to SAT suv mean; Table S2: Adverse effects according to VAT suv mean.
