# Supplementary material for: Impact of Body Composition on Progression-Free Survival in Patients with Metastatic Breast Cancer Treated with Ribociclib
Source: Curr Oncol. 2025 Sep 13;32(9):510. doi: 10.3390/curroncol32090510 (PMC12468890; doi:10.3390/curroncol32090510)
Supplement: Supplementary file 1 [file curroncol-32-00510-s001.zip › curroncol-3804202-supplementary.pdf]

Table S1. Adverse effects according to SAT suv mean.

	**SAT suv mean ≥ 27 (n:39)**	**SAT suv mean < 27 (n:34)**	***p***
Neutropenia, n (%)	7 (17.9%)	13 (38.2%)	0.053
Increased liver function tests, n (%)	-	1 (2.9%)	
Qtc prolonged, n (%)	-	5 (14.7%)	
Nephrotoxicity, n (%)	-	1 (2.9%)	
Pneumonitis, n (%)	-	1 (2.9%)	
Skin toxicity, n (%)	2 (5.1%)	-	

Table S2. Adverse effects according to VAT suv mean.

	**VAT suv mean ≥ 0.72 (n:39)**	**SAT suv mean < 0.72 (n:34)**	***p***
Neutropenia, n (%)	7 (17.9%)	13 (38.2%)	0.053
Increased liver function tests, n (%)	2 (5.1%)	1 (2.9%)	0.639
Qtc prolonged, n (%)	1 (2.6%)	4 (11.8%)	0.121
Nephrotoxicity, n (%)	1 (2.6%)	-	
Pneumonitis, n (%)	-	1 (2.9%)	
Skin toxicity, n (%)	1 (2.6%)	1 (2.9%)	
